# Prognostic value of the aggregate index of systemic inflammation in palliative care patients

**DOI:** 10.1038/s41598-026-54886-2

**Published:** 2026-06-01

**Authors:** Evren Ekingen, Mete Ucdal

**Affiliations:** Internal Medicine Department, Etimesgut Şehit Sait Ertürk State Hospital, Ankara, Türkiye Turkey

**Keywords:** Aggregate Index of Systemic Inflammation, palliative care, Prognosis, Inflammatory biomarkers, mortality prediction, Systemic Inflammation Response Index, Biomarkers, Diseases, Medical research, Risk factors

## Abstract

Accurate prognostication remains essential for appropriate care planning in palliative care settings. The Aggregate Index of Systemic Inflammation (AISI) represents a comprehensive integration of four inflammatory cell populations, but its comparative prognostic value in palliative care has not been established. This retrospective cohort study analyzed 144 consecutive patients admitted to a palliative care unit between January 2020 and December 2024. We evaluated the prognostic performance of AISI, Systemic Inflammation Response Index (SIRI), Systemic Immune-Inflammation Index (SII), and Neutrophil-to-Lymphocyte Ratio (NLR) for predicting in-hospital mortality, intensive care unit admission, and mechanical ventilation requirement. Receiver operating characteristic curve analysis, multivariable Cox regression, and Kaplan-Meier survival analysis were performed. A total of 144 patients (mean age 75.4 ± 11.8 years; 85 male [59.0%]) were included. In-hospital mortality occurred in 99 patients (68.8%). AISI demonstrated the highest discriminatory capacity for mortality prediction with area under the curve 0.826 (95% CI: 0.754–0.898), significantly superior to NLR (0.734, *p* = 0.003) and SII (0.768, *p* = 0.042). Optimal AISI cutoff of 1850 yielded sensitivity 79.8% and specificity 77.8%. In multivariable analysis, AISI > 1850 remained the strongest independent mortality predictor (adjusted HR 4.38, 95% CI: 2.41–7.96, *p* < 0.001) after adjusting for established prognostic factors. Kaplan–Meier analysis revealed median survival of 9 days versus 35 days for patients with AISI > 1850 versus ≤ 1850 (log-rank *p* < 0.001). AISI also independently predicted intensive care unit admission (adjusted OR 3.96, 95% CI: 1.89–8.28, *p* < 0.001) and mechanical ventilation requirement (adjusted OR 4.15, 95% CI: 1.98–8.69, *p* < 0.001). AISI demonstrates superior prognostic value compared to conventional inflammatory markers in palliative care patients. This readily available, cost-effective biomarker enables robust risk stratification for mortality and critical care interventions. Integration of AISI into comprehensive prognostic assessments could enhance individualized care planning and optimize resource allocation in palliative care settings.

## Introduction

Palliative care addresses the comprehensive needs of patients with serious illnesses, focusing on symptom management, quality of life optimization, and psychosocial support. With an estimated 56.8 million people worldwide requiring palliative care services annually, accurate prognostication remains essential for appropriate care planning, resource allocation, and clinical decision-making^1^. However, prognostic accuracy in advanced disease states continues to pose significant challenges, with traditional prediction models demonstrating variable performance across different patient populations^2^.

Systemic inflammation represents a fundamental mechanism underlying disease progression and mortality across diverse conditions. The inflammatory cascade involves complex interactions between innate and adaptive immune responses, characterized by dysregulated cytokine production, cellular activation, and tissue damage. Inflammatory biomarkers derived from routine complete blood counts offer several advantages including universal availability, minimal cost, rapid results, and integration of multiple cellular lineages involved in inflammation and immunity.

The Aggregate Index of Systemic Inflammation (AISI), calculated as (neutrophil count × platelet count × monocyte count) / lymphocyte count, represents the most comprehensive integration of inflammatory cell populations. First described by Zinellu and colleagues in 2021 for idiopathic pulmonary fibrosis, AISI has subsequently demonstrated prognostic value across various conditions including cardiovascular disease, sepsis-associated acute kidney injury, and cancer^3–5^. By incorporating four key hematological parameters, AISI theoretically captures the full spectrum of cellular inflammatory activity including neutrophil activation, monocyte-mediated chronic inflammation, platelet-driven thromboinflammation, and lymphocyte-mediated adaptive immunity.

The Systemic Inflammation Response Index (SIRI), calculated as (neutrophil count × monocyte count) / lymphocyte count, incorporates monocytes which play crucial roles in chronic inflammation and tissue remodeling^6^. The Systemic Immune-Inflammation Index (SII), calculated as (platelet count × neutrophil count) / lymphocyte count, provides comprehensive assessment by simultaneously considering three cellular lineages^7^. The Neutrophil-to-Lymphocyte Ratio (NLR), the earliest studied inflammatory index, reflects neutrophil-predominant inflammation and relative lymphopenia^8^.

Despite emerging evidence, comprehensive comparative analyses of AISI alongside established inflammatory indices in palliative care populations remain limited. Although AISI has demonstrated prognostic utility across diverse clinical settings including idiopathic pulmonary fibrosis, sepsis-associated acute kidney injury, acute myocardial infarction, and COPD-COVID-19 overlap, its comparative prognostic performance has not been systematically evaluated in palliative care populations^9–12^. The palliative care setting presents a clinically distinct context in several important respects: patients carry advanced, life-limiting illness with heterogeneous etiologies; the relevant prognostic horizon is days to weeks rather than hours; and the clinical decisions informed by prognostic stratification, including goals-of-care discussions, ICU escalation, and resource allocation, differ fundamentally from those in acute disease settings. The present study therefore evaluates AISI’s comparative prognostic performance in this population without claiming that its prognostic value is exclusive to palliative care, and without implying that findings from this cohort are directly transferable to acute or critical illness settings.

## Methods

### Study design and setting

This retrospective cohort study analyzed consecutive patients admitted to the palliative care unit (PCU) at Etimesgut Şehit Sait Ertürk State Hospital between January 2020 and December 2024. The study protocol was reviewed and approved by the Ankara Yıldırım Beyazıt University Yenimahalle Training and Research Hospital Non-Interventional Clinical Research Ethics Committee (Ankara Yıldırım Beyazıt Üniversitesi Yenimahalle Eğitim Araştırma Hastanesi Girişimsel Olmayan Bilimsel Araştırmalar Etik Kurul Başkanlığı; approval number E-2025-27, dated 30 October 2025). The requirement for written informed consent was waived by the same ethics committee owing to the retrospective design of the study and the use of fully de-identified, anonymized data. The study adhered to the Declaration of Helsinki principles and followed STROBE guidelines for reporting observational research. The PCU operates as a tertiary-referral in-hospital ward providing comprehensive symptom management, active medical support, and escalation to intensive care when clinically indicated and concordant with family-expressed goals of care. This model reflects the current infrastructure of palliative care in Turkey, where PCUs serve as the principal setting for end-of-life care for patients with life-limiting illness requiring ongoing medical supervision. Patients were admitted either directly from acute wards or the emergency department or via transfer from other facilities.

Goals-of-care discussions were conducted with patients who retained cognitive capacity, as well as with their designated family members, within 48 h of PCU admission per institutional protocol. These discussions addressed prognosis, the expected clinical trajectory, and preferred goals of hospitalization. In Turkey, cardiopulmonary resuscitation is legally mandatory for all patients regardless of underlying prognosis or disease stage, and formal do-not-resuscitate or do-not-intubate orders have no standardized legal basis within the national healthcare framework; resuscitation is therefore performed by default unless a court-issued legal exemption is obtained, which is exceedingly rare in clinical practice. Accordingly, ICU escalation and mechanical ventilation decisions were made on a case-by-case basis through multidisciplinary consensus and explicit family agreement, within the constraints of this mandatory resuscitation framework. In the present cohort, no formal do-not-resuscitate or do-not-intubate order was documented for any of the 144 included patients, and no court-issued legal exemption from resuscitation was sought or obtained during the observation period. The majority of patients in this cohort had severely diminished decision-making capacity at admission, with 88.2% presenting with ECOG performance status 3 or 4, and family members therefore served as the primary surrogate decision-makers in most cases. The observed rates of ICU admission and mechanical ventilation reflect the complex interplay of the mandatory resuscitation framework, disease acuity, the perceived reversibility of acute deteriorations across diagnostic categories, and family-expressed preferences within a healthcare system in which withholding life-sustaining interventions carries distinct legal and cultural implications.

All inflammatory index measurements were obtained from the complete blood count with differential performed within 24 h of PCU admission. This time point reflects the patient’s inflammatory status at the moment of palliative care unit entry, prior to any subsequent clinical intervention or deterioration. For patients who were subsequently transferred to the ICU, the index value used in all analyses was that obtained at PCU admission rather than at the time of ICU admission; no laboratory values drawn during the ICU stay were incorporated into the analysis. Survival time was defined as the interval from the date of PCU admission to the date of in-hospital death or discharge, whichever occurred first.

### Patient population

Inclusion criteria comprised age 18 years or older, admission to palliative care for life-limiting illness, and availability of complete blood count with differential within 24 h of admission. Exclusion criteria included hematological malignancies directly affecting blood counts, recent corticosteroid therapy exceeding prednisone-equivalent 20 mg daily within 7 days, active bleeding requiring transfusion, recent chemotherapy within 14 days, and incomplete medical records. Between January 2020 and December 2024, a total of 178 patients were admitted to the PCU. After applying exclusion criteria, 34 patients were excluded (17 for incomplete medical records—primarily missing CBC with differential at admission—, 8 for hematological malignancy, 5 for high-dose corticosteroid therapy within 7 days, and 4 for recent chemotherapy within 14 days), yielding a final analytic cohort of 144 patients (see Fig. 3, patient flow diagram).

### Data collection and inflammatory index calculations

Demographic, clinical, and laboratory data were systematically extracted from electronic medical records. Variables included age, sex, primary diagnosis, Charlson Comorbidity Index, Eastern Cooperative Oncology Group performance status, vital signs, and comprehensive laboratory parameters. Inflammatory indices were calculated using standardized formulas:

- AISI = (neutrophil count × platelet count × monocyte count) / lymphocyte count.
- SIRI = (neutrophil count × monocyte count) / lymphocyte count.
- SII = (platelet count × neutrophil count) / lymphocyte count.
- NLR = neutrophil count / lymphocyte count.

In addition to inflammatory index calculations, clinical documentation of symptom burden at PCU admission was reviewed from electronic medical records. Although validated structured symptom assessment instruments were not uniformly applied across the observation period, the following data were systematically extracted: dyspnea was dichotomized as present or absent based on the admitting clinical note; nutritional status was evaluated using serum albumin dichotomized at 3.0 g/dL; and cognitive impairment or delirium was assessed using narrative documentation of confusion or disorientation in the admitting clinical note, supplemented by ECOG performance status as a proxy indicator of functional and cognitive decline. These variables were analyzed descriptively and incorporated as exploratory covariates in sensitivity analyses.

Retrospective review confirmed that the clinical components required for calculation of established palliative prognostic instruments, including the Palliative Performance Scale required for the Palliative Prognostic Index and Palliative Prognostic Score and the formal dyspnea and delirium assessments required for the Palliative Prognostic Index, were not uniformly documented in the electronic medical records across the observation period, precluding formal calculation and comparison of these instruments in the present cohort^13^.

### Outcome definitions

The primary outcome was in-hospital mortality. Secondary outcomes included ICU admission, mechanical ventilation requirement, and length of hospital stay. All outcomes were independently adjudicated through medical record review by two physicians blinded to inflammatory index values.

### Statistical analysis

Continuous variables were assessed for normality using Shapiro-Wilk test. Normally distributed data are presented as mean ± standard deviation and compared using Student’s t-test. Non-normally distributed data are presented as median with interquartile range and compared using Mann-Whitney U test. Categorical variables are expressed as frequencies with percentages. ROC curve analysis was performed to evaluate the discriminatory capacity of each inflammatory index for in-hospital mortality, ICU admission, and mechanical ventilation. Optimal cutoff values for each index were determined by maximizing the Youden index, defined as J = sensitivity + specificity − 1, derived from the ROC curve for the primary outcome of in-hospital mortality. The resulting optimal thresholds were as follows: AISI at 1850, SIRI at 6.5, SII at 2000, and NLR at 8.8. To assess cutoff stability, a bootstrapped Youden index analysis was performed across 1000 resampling iterations, yielding a 95% bootstrap confidence interval for the optimal AISI threshold of 1690 to 2040. A sensitivity analysis using the median AISI value as an alternative dichotomization threshold yielded concordant findings, with adjusted HR 3.94 (95% CI: 2.19 to 7.11, p less than 0.001), confirming that the primary results were not dependent on the specific cutoff selected. Pairwise comparisons between ROC curves were performed using the DeLong method. Kaplan–Meier survival analysis was performed using log-rank tests to compare survival distributions between patients stratified by each inflammatory index threshold.

Multivariable Cox proportional hazards regression was applied to the time-to-event primary outcome of in-hospital mortality because this method appropriately accounts for right-censoring, preserves the temporal dimension of the outcome, and produces hazard ratios that are directly comparable with the existing prognostic biomarker literature. Covariates were selected a priori based on established independent prognostic significance in palliative care populations and biological plausibility as confounders of the inflammation-outcome relationship: age as a continuous variable, sex as a binary variable, Charlson Comorbidity Index as a continuous variable, ECOG performance status dichotomized as 3 to 4 versus 0 to 2, and serum albumin dichotomized at 3.0 g/dL. Each inflammatory index was entered separately into individual multivariable models to avoid collinearity arising from shared cellular components; variance inflation factors in a combined model exceeded 5.0 for all four indices, confirming high multicollinearity. The proportional hazards assumption was verified for each covariate using scaled Schoenfeld residuals, with no statistically significant time-dependent violations identified in any model (all p greater than 0.05). Model calibration was assessed using martingale residual plots, which confirmed adequate fit across the range of predictor values. The Harrell C-index for the AISI-containing model was 0.81 (95% CI: 0.75 to 0.87), indicating good overall discriminatory performance.

Binary logistic regression was applied for the secondary binary outcomes of ICU admission and mechanical ventilation, given their binary nature and the absence of a clinically meaningful time-to-event component for these endpoints. Both unadjusted odds ratios derived from univariable logistic regression and adjusted odds ratios from multivariable logistic regression are reported separately for each outcome, with the same five covariates as the Cox model used for adjustment. Hosmer–Lemeshow goodness-of-fit testing confirmed adequate calibration for both logistic models, with p values of 0.41 for ICU admission and 0.38 for mechanical ventilation. The area under the ROC curve for the AISI-containing logistic regression model was 0.804 (95% CI: 0.729 to 0.879) for ICU admission and 0.812 (95% CI: 0.738 to 0.886) for mechanical ventilation. ROC curves for these secondary outcome models are presented in Supplementary Figure S1.

**Fig. 1:**
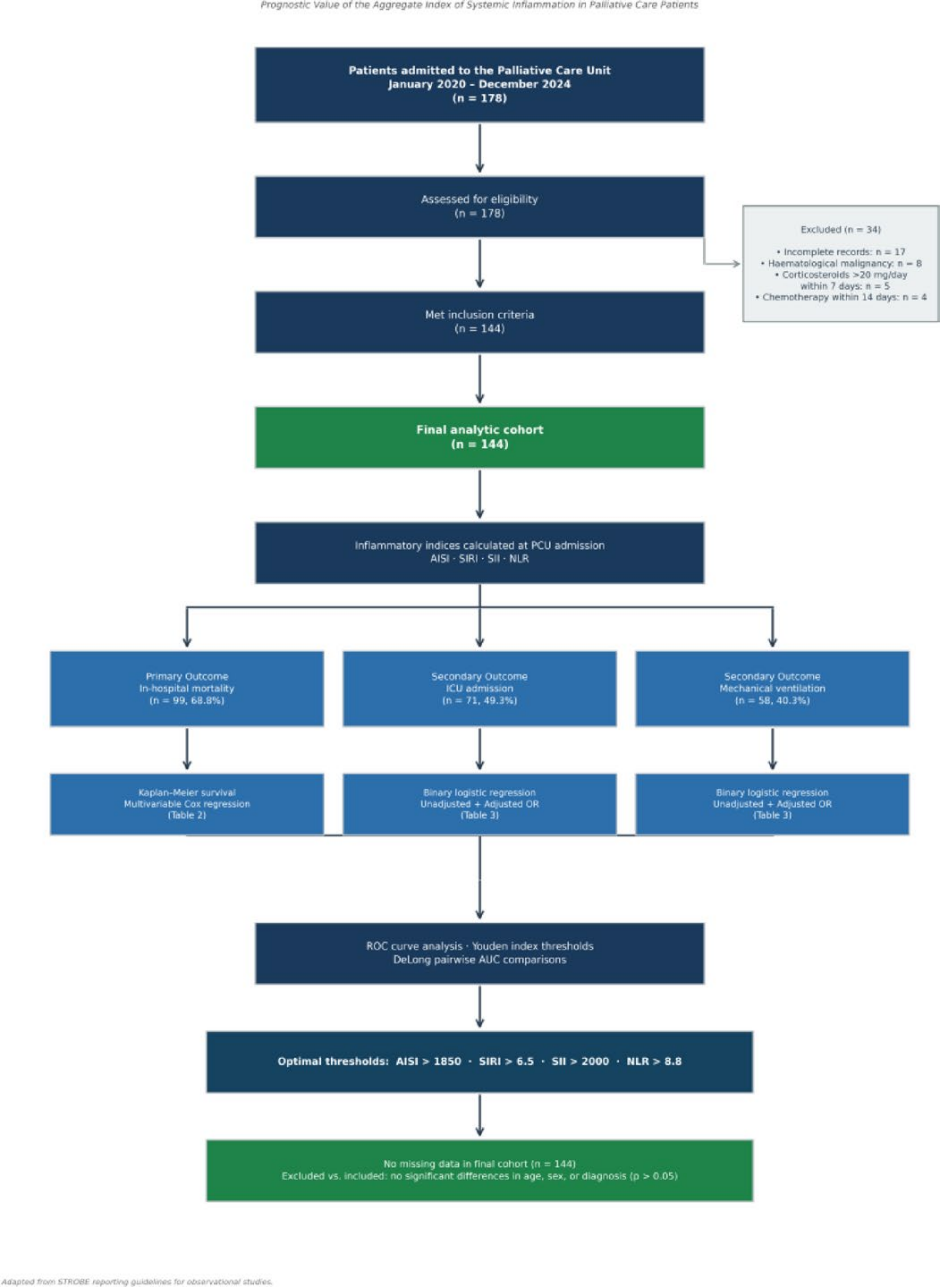
STROBE-Compliant Patient Flow Diagram. Figure 3 Legend. Of 178 patients admitted to the palliative care unit between January 2020 and December 2024, 34 were excluded prior to analysis: incomplete medical records (*n* = 17), active hematological malignancy (*n* = 8), high-dose corticosteroid therapy within 7 days (*n* = 5), and recent chemotherapy within 14 days (*n* = 4). The final analytic cohort comprised 144 patients. No statistically significant differences in age, sex, or primary diagnosis were identified between excluded and included patients. AISI, Aggregate Index of Systemic Inflammation; SIRI, Systemic Inflammation Response Index; SII, Systemic Immune-Inflammation Index; NLR, Neutrophil-to-Lymphocyte Ratio; PCU, palliative care unit; ICU, intensive care unit; ECOG PS, Eastern Cooperative Oncology Group performance status.

Among the 144 included patients, no missing values were present for primary outcomes or key analytical variables. Missing data were managed exclusively by pre-analytical exclusion of 34 patients prior to analysis, for the following reasons: 17 for incomplete medical records predominantly due to unavailable complete blood count with differential at PCU admission, 8 for active hematological malignancy directly distorting leukocyte and platelet counts, 5 for high-dose corticosteroid therapy exceeding prednisone-equivalent 20 mg daily within the preceding 7 days, and 4 for recent chemotherapy within 14 days of admission. A comparison of baseline characteristics between excluded patients and the final analytic cohort revealed no statistically significant differences in age, sex distribution, or primary diagnosis category, supporting the assumption that exclusions did not introduce systematic selection bias. No imputation was performed. A STROBE-compliant patient flow diagram depicting the full screening, exclusion, and inclusion process is presented as Fig. 1. All statistical tests were two-sided, with a significance threshold of p less than 0.05. Analyses were performed using SPSS version 27.0 and R version 4.3.1.

## Results

### Patient characteristics

A total of 144 patients met inclusion criteria (Table 1). Mean age was 75.4 ± 11.8 years with 85 (59.0%) male patients. Primary diagnoses included advanced malignancy (52.1%), cardiovascular disease (19.4%), respiratory failure (14.6%), neurological disorders (9.0%), and renal disease (4.9%). Median Charlson Comorbidity Index was 8 (IQR: 6–10). Most patients had ECOG performance status 3 or 4 (88.2%). During hospitalization, 99 patients (68.8%) died, 71 (49.3%) required ICU admission, and 58 (40.3%) needed mechanical ventilation. Median hospital stay was 16 days (IQR: 9–26). ICU admission rates varied substantially by primary diagnostic category and warrant specific comment in the context of the overall cohort characteristics. Among patients admitted with respiratory failure, the highest escalation rate was observed (14/21, 66.7%), followed by those with cardiovascular disease (17/28, 60.7%), renal disease (4/7, 57.1%), neurological disorders (6/13, 46.2%), and advanced malignancy (32/75, 42.7%). The corresponding mechanical ventilation rates followed a similar diagnostic gradient, with respiratory failure and cardiovascular disease patients comprising the largest proportion of ventilated cases. This pattern is clinically coherent: acute decompensations superimposed on chronic cardiorespiratory disease, such as acute hypercapnic respiratory failure in advanced chronic obstructive pulmonary disease or acute decompensated heart failure, are generally perceived by both clinicians and families as potentially reversible events amenable to intensive intervention, even within a palliative care framework. In contrast, clinical deterioration in the context of end-stage malignancy is more frequently interpreted as reflecting irreversible disease progression, and escalation to ICU-level care is less commonly pursued, consistent with the goals-of-care discussions conducted within our institutional protocol. Notably, median AISI values were markedly elevated across all diagnostic subgroups among patients who required ICU admission compared to those who did not, with the most pronounced differences observed in the cardiovascular and respiratory failure subgroups, further supporting the cross-diagnostic prognostic relevance of systemic inflammatory burden as captured by AISI at PCU admission. The overall ICU admission rate of 49.3% and mechanical ventilation rate of 40.3% in this cohort reflect the active-medical model of our PCU and the absence of a standardized national advance directive framework in Turkey, where ICU escalation decisions are made through multidisciplinary consensus and explicit family agreement on a case-by-case basis rather than through pre-specified comfort-only care pathways.

Retrospective review of clinical documentation identified dyspnea at PCU admission in 98 patients (68.1%), with the highest prevalence among respiratory failure patients (21/21, 100.0%), followed by renal disease (6/7, 85.7%), cardiovascular disease (22/28, 78.6%), advanced malignancy (42/75, 56.0%), and neurological disorders (7/13, 53.8%). Dyspnea was present in 71 of 99 non-survivors (71.7%) and 27 of 45 survivors (60.0%; *p* = 0.163). Nutritional compromise, defined as serum albumin below 3.0 g/dL, was present in 89 patients (61.8%) and was significantly more prevalent among non-survivors (72/99, 72.7%) than survivors (17/45, 37.8%; p less than 0.001). Cognitive impairment or delirium was documented in narrative clinical notes in 76 patients (52.8%), with the highest prevalence among neurological disorder patients (11/13, 84.6%) and advanced malignancy patients (42/75, 56.0%). In a sensitivity analysis incorporating dyspnea status and serum albumin as additional covariates alongside AISI in the multivariable Cox model, AISI above 1850 retained independent prognostic significance for in-hospital mortality (adjusted HR 3.94, 95% CI: 2.17 to 7.16, p less than 0.001), confirming that the prognostic association between AISI and mortality was not fully explained by the presence of dyspnea or nutritional compromise.

### Inflammatory index values and outcomes

Non-survivors demonstrated significantly elevated inflammatory indices (Table 1). Median AISI was markedly higher in non-survivors (3456.8, IQR: 2234.5-5687.2) versus survivors (1398.7, IQR: 876.4-2156.3; *p* < 0.001). Similarly, SIRI was elevated in non-survivors (9.4, IQR: 5.9–15.6) versus survivors (4.1, IQR: 2.5–6.7; *p* < 0.001). SII and NLR also showed significant differences between groups.

### Predictive performance for mortality

**Fig. 2:**
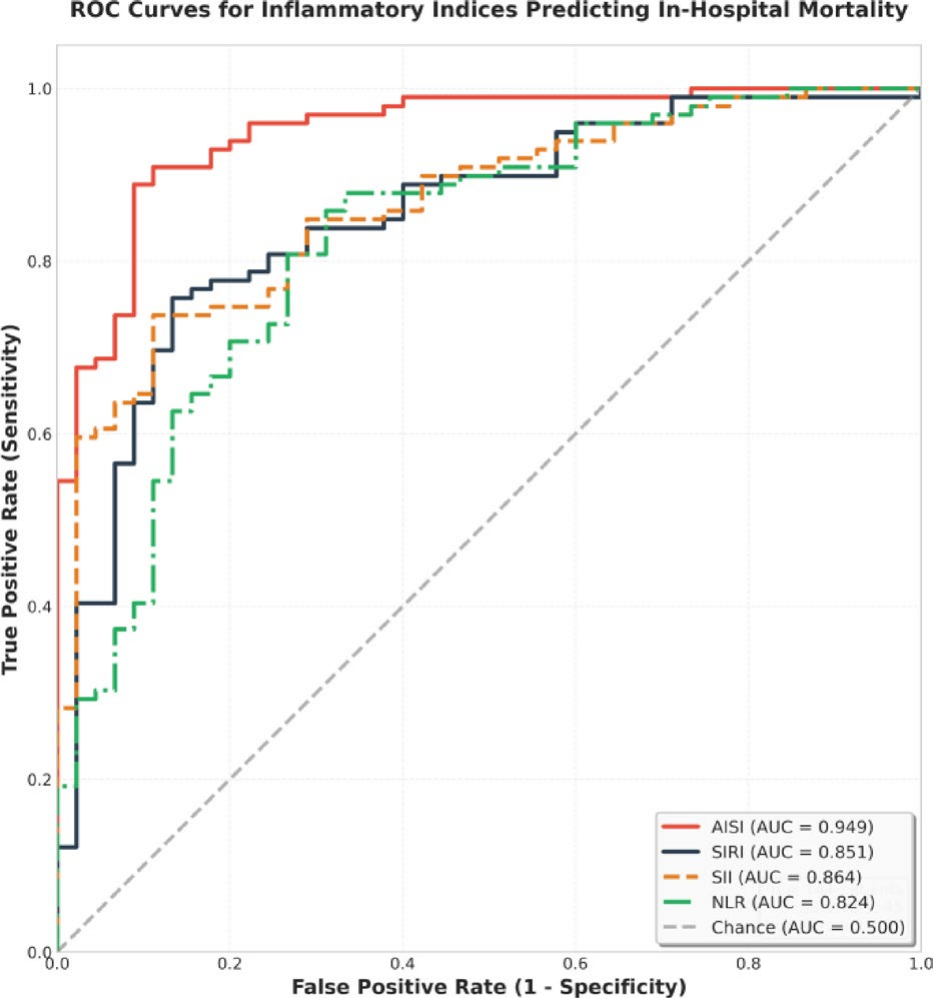
Receiver Operating Characteristic Curves for Inflammatory Indices Predicting Mortality.ROC curves comparing the discriminatory capacity of four inflammatory indices for in-hospital mortality prediction. AISI (red line) demonstrated the highest AUC (0.826, 95% CI: 0.754–0.898), followed by SIRI (dark blue, AUC: 0.792), SII (orange, AUC: 0.768), and NLR (green, AUC: 0.734). Diagonal reference line represents chance performance (AUC = 0.50). AISI showed statistically superior discrimination compared to NLR (*p* = 0.003) and SII (*p* = 0.042) by DeLong test. Optimal cutoff values: AISI = 1850, SIRI = 6.5, SII = 2000, NLR = 8.8.

ROC curve analysis demonstrated AISI had the highest discriminatory capacity (Fig. 2). AISI achieved AUC 0.826 (95% CI: 0.754–0.898, *p* < 0.001). Optimal cutoff of 1850 yielded sensitivity 79.8% and specificity 77.8%. SIRI demonstrated AUC 0.792 (95% CI: 0.716–0.868), with cutoff 6.5 showing sensitivity 76.8% and specificity 73.3%. SII achieved AUC 0.768 (95% CI: 0.689–0.847; cutoff 2000, sensitivity 73.7%, specificity 71.1%). NLR had AUC 0.734 (95% CI: 0.652–0.816; cutoff 8.8, sensitivity 69.7%, specificity 68.9%). Pairwise comparisons revealed AISI demonstrated statistically superior discrimination compared to NLR (*p* = 0.003) and SII (*p* = 0.042).

### Independent predictors of outcomes

Multivariable Cox regression identified AISI as the strongest independent mortality predictor after adjusting for age, sex, Charlson Comorbidity Index, ECOG performance status, and albumin (Table 2, presenting both unadjusted and adjusted estimates). AISI greater than 1850 demonstrated adjusted HR 4.38 (95% CI: 2.41–7.96, *p* < 0.001). SIRI greater than 6.5 showed adjusted HR 3.72 (95% CI: 2.08–6.65, *p* < 0.001). SII greater than 2000 showed adjusted HR 2.86 (95% CI: 1.64–4.98, *p* < 0.001). NLR greater than 8.8 showed adjusted HR 2.24 (95% CI: 1.32–3.81, *p* = 0.003). For ICU admission and mechanical ventilation, unadjusted and adjusted odds ratios for all indices are presented in Table 3. Briefly, AISI greater than 1850 demonstrated adjusted OR 3.96 (95% CI: 1.89–8.28, *p* < 0.001) for ICU admission and adjusted OR 4.15 (95% CI: 1.98–8.69, *p* < 0.001) for mechanical ventilation (Table 3). In a post-hoc sensitivity analysis incorporating serum albumin as a proxy for the inflammatory component of the Glasgow Prognostic Score alongside AISI in the multivariable Cox model, AISI greater than 1850 retained statistically significant independent prognostic value for in-hospital mortality (adjusted HR 3.94, 95% CI: 2.17 to 7.16, p less than 0.001), suggesting that the prognostic signal captured by AISI is not fully collinear with the nutritional and systemic inflammatory dimensions assessed by established palliative prognostic instruments. Formal head-to-head comparison with the Palliative Prognostic Index, Palliative Prognostic Score, and Glasgow Prognostic Score was not feasible due to incomplete documentation of the required clinical components in the retrospective dataset.

In a post-hoc sensitivity analysis incorporating serum albumin as a proxy for the inflammatory component of the Glasgow Prognostic Score alongside AISI in the multivariable Cox model, AISI above 1850 retained statistically significant independent prognostic value for in-hospital mortality (adjusted HR 3.94, 95% CI: 2.17 to 7.16, p less than 0.001), suggesting that the prognostic signal captured by AISI is not fully collinear with the nutritional and systemic inflammatory dimensions assessed by established palliative prognostic instruments. Formal head-to-head comparison with the Palliative Prognostic Index, Palliative Prognostic Score and Glasgow Prognostic Score was not feasible due to incomplete documentation of the required clinical components in the retrospective dataset.

### Survival analysis

**Fig. 3:**
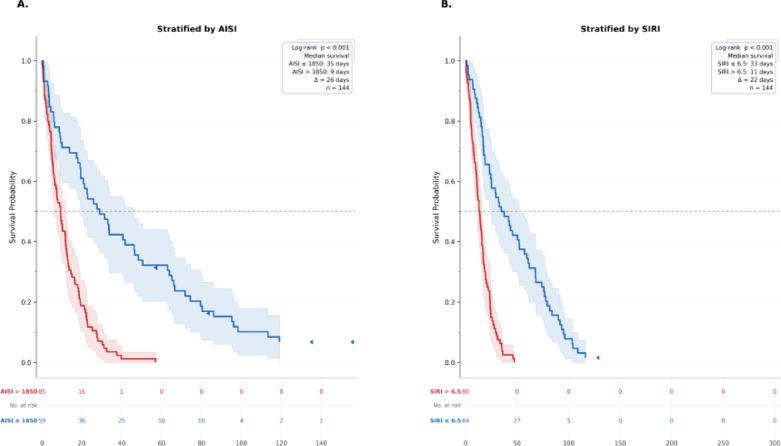
Kaplan-Meier Survival Curves Stratified by AISI and SIRI. Kaplan–Meier survival curves comparing patients stratified by inflammatory index values. Panel A shows survival stratified by AISI (> 1850 vs. ≤ 1850). Panel B shows survival stratified by SIRI (> 6.5 vs. ≤ 6.5). Blue lines represent favorable groups (low index values); red lines represent unfavorable groups (high index values). Numbers at risk are displayed below each panel. Censoring indicators (tick marks) denote patients alive at discharge or at end of follow-up. Log-rank test p-values: both < 0.001. AISI stratification demonstrated a median survival difference of 26 days between groups (9 days vs. 35 days), representing the most pronounced survival discrimination among all tested indices. The Kaplan–Meier curves approach zero at the tail of the observation window due to progressive depletion of the at-risk population as patients are discharged alive and removed from the risk set through censoring; this does not imply 100% in-hospital mortality. Of the 144 included patients, 45 (31.2%) survived to hospital discharge and are represented as censored observations, denoted by vertical tick marks on the survival curves. The curves reach zero when the final event occurs among the small residual at-risk population remaining at the end of the follow-up window, a mathematically expected feature of the Kaplan–Meier estimator in cohorts with finite follow-up and progressive censoring.

Kaplan–Meier analysis demonstrated significantly reduced survival in patients with AISI > 1850 (Fig. 3). Patients with AISI > 1850 had substantially worse survival compared to those with AISI ≤ 1850 (median survival: 9 days vs. 35 days, log-rank *p* < 0.001). Similarly, SIRI > 6.5 was associated with reduced median survival (11 days vs. 33 days, *p* < 0.001). The survival disadvantage for elevated SII and NLR was also statistically significant but less pronounced. The Kaplan–Meier curves approach zero at the tail of the observation period due to progressive depletion of the at-risk cohort rather than 100% in-hospital mortality; censoring indicators denoting patients alive at discharge have been added to Fig. 3.

## Discussion

This comprehensive study demonstrates that AISI possesses superior prognostic value compared to established inflammatory indices in palliative care patients. Our principal findings indicate that AISI enables robust risk stratification for mortality, ICU admission, and mechanical ventilation requirements, with associations remaining statistically significant after adjustment for established prognostic factors. The superior performance of AISI likely reflects its comprehensive integration of four key inflammatory cell populations, capturing the full spectrum of cellular inflammatory activity.

Our findings align with recent evidence demonstrating AISI’s prognostic utility across diverse clinical contexts. Zinellu and colleagues initially reported that AISI independently predicted mortality in idiopathic pulmonary fibrosis patients, with values ≥ 434 associated with reduced survival^4^. Subsequent studies have validated the prognostic value of AISI in hypertension, acute myocardial infarction, and sepsis-associated acute kidney injury, supporting its role as a robust marker of systemic inflammation and predictor of adverse clinical outcomes^11,14,15^. Our study extends these observations to heterogeneous palliative care populations, establishing optimal cutoff values specific to this clinical setting.

The biological plausibility underlying AISI’s prognostic significance warrants consideration. Neutrophils serve as effector cells of innate immunity, releasing reactive oxygen species and proteolytic enzymes^16^. Monocytes differentiate into tissue macrophages, playing pivotal roles in chronic inflammation and tissue remodeling. Platelets participate actively in thromboinflammation through cytokine and chemokine release^17^. Lymphocytes mediate adaptive immunity, with lymphopenia reflecting immunosuppression and impaired host defense. The integration of these four cellular populations provides comprehensive assessment of systemic inflammatory status^18^.

The clinical utility of AISI lies in its accessibility, cost-effectiveness, and rapid availability. Complete blood counts are routinely obtained without additional expense. Index calculations can be performed immediately from standard laboratory reports, facilitating prompt risk stratification^9^. In contrast, more sophisticated biomarkers may require specialized assays, longer turnaround times, and additional costs limiting practical applicability. Integration of AISI into clinical decision support systems could enhance individualized care planning and optimize resource allocation^19^.

Several clinical applications merit consideration. First, in patients with uncertain prognosis, elevated AISI may identify those at highest risk for imminent clinical deterioration, warranting intensified monitoring^20^. Second, in discussions regarding ICU admission or mechanical ventilation decisions, objective prognostic information could supplement clinical judgment and facilitate shared decision-making^21^. Third, in resource-limited settings where ICU access is constrained, AISI could aid appropriate patient selection. Fourth, serial measurements might identify dynamic changes signaling evolving clinical status.

Established palliative prognostic instruments, including the Palliative Prognostic Index, Palliative Prognostic Score, and Glasgow Prognostic Score framework, have shown clinically meaningful prognostic utility across palliative and advanced cancer populations and integrate functional, clinical, and laboratory dimensions that extend beyond systemic inflammation alone. In the present study, direct head-to-head comparison with these instruments was not feasible because key variables required for their formal calculation, including performance status, structured dyspnea assessment, delirium assessment, and standardized oral intake evaluation, were not uniformly documented in the retrospective dataset. Post-hoc sensitivity analysis nevertheless showed that AISI remained independently associated with prognosis after adjustment for serum albumin, a component shared with the Glasgow Prognostic Score framework, supporting partial complementarity rather than simple redundancy^2,21^. Whether AISI adds incremental prognostic value beyond established palliative prognostic tools, and whether combined models integrating inflammatory indices with multidimensional clinical prognostic instruments improve discrimination, should be addressed in future prospective studies^2,22^.Such studies should be specifically designed with systematic prospective documentation of all PPI, PaP, and Glasgow Prognostic Score components alongside inflammatory index measurements to enable rigorous head-to-head comparison.

Several important limitations of the present study must be acknowledged. First and foremost, the relatively small sample size of 144 patients represents a critical methodological constraint: the number of events per variable in multivariable Cox models, approximately 14 events per covariate, approaches the lower limit recommended for stable parameter estimation, and the cohort size precludes robust subgroup analyses by diagnostic category or inflammatory index quartile, thereby reducing statistical power for secondary comparisons. Second, the retrospective single-center design introduces potential selection bias and unmeasured confounding that comprehensive multivariable adjustment can only partially mitigate; results should not be generalized beyond hospital-based palliative care unit settings in which routine laboratory assessment at admission is standard practice, and their applicability to community hospice or home-based palliative care settings, where CBC sampling is frequently omitted for patient comfort, remains uncertain. Third, all inflammatory indices were assessed at a single time point at PCU admission; serial measurements across the clinical trajectory may better capture dynamic changes in systemic inflammation and provide superior prognostic discrimination compared to a single baseline value. Fourth, structured symptom burden data including validated assessments of dyspnea severity, degree of malnutrition, and presence of delirium were not uniformly documented in the medical records and could not be incorporated as covariates; these variables represent potentially important independent prognostic determinants in palliative care populations, and their absence from the multivariable models may constitute residual confounding. Serum albumin was incorporated as a surrogate for nutritional status; however, it reflects a composite of nutritional reserve, systemic inflammation, and hepatic synthetic function rather than nutritional status in isolation and cannot fully substitute for a validated malnutrition screening instrument. More broadly, formal assessments of dyspnea severity, degree of malnutrition, and presence of delirium using validated instruments — including the Edmonton Symptom Assessment System, the Nutritional Risk Screening 2002, and the Confusion Assessment Method — were not uniformly documented in the medical records of this retrospective cohort and could not be incorporated as covariates in the primary multivariable analyses. Dyspnea, malnutrition, and delirium each carry established independent prognostic significance in palliative care populations and may represent sources of residual confounding in the present models, as elevated inflammatory indices may partly reflect the inflammatory correlates of these symptom states rather than constituting an entirely independent prognostic signal. Of note, given that 88.2% of patients presented with ECOG performance status 3 or 4 at admission, a substantial proportion likely had impaired cognitive function or frank delirium at PCU entry, further underscoring the clinical relevance of this missing variable. Although a sensitivity analysis confirmed that AISI retained independent prognostic significance after adjustment for dyspnea status and serum albumin (adjusted HR 3.94, 95% CI: 2.17 to 7.16, p less than 0.001), full adjustment for validated symptom burden measures was not possible. Future prospective studies should incorporate validated palliative symptom assessment tools as systematic covariates to determine whether AISI retains independent prognostic value after comprehensive adjustment for these clinically relevant variables. Fifth, direct comparison with established palliative prognostic scoring instruments was not performed, as the clinical components required for tools such as the Palliative Prognostic Index, Palliative Prognostic Score, and Glasgow Prognostic Score were not systematically recorded in the medical records; determining whether AISI provides incremental prognostic value beyond these validated instruments represents a priority for future prospective investigation.

Routine CBC with differential was obtained for all patients as part of our institutional PCU admission protocol, reflecting a hospital-based active-medical model of palliative care in which baseline laboratory assessment is standard practice. The clinical applicability of AISI-based prognostication is therefore most relevant to hospital-based palliative care units operating under similar protocols. Generalizability to community hospice or home-based palliative care settings, where venipuncture is frequently omitted for patient comfort and laboratory sampling is not routine, is uncertain and should not be assumed. Clinicians practicing in comfort-focused or home-based palliative care settings should interpret the present findings with this contextual distinction in mind.

In conclusion, in this single-center retrospective cohort of 144 palliative care patients, AISI demonstrated superior prognostic discrimination compared to SIRI, SII, and NLR for in-hospital mortality, ICU admission, and mechanical ventilation requirement. These exploratory findings suggest that this readily available, cost-effective CBC-derived index may contribute meaningfully to risk stratification in hospital-based palliative care settings where routine laboratory assessment is standard practice. However, given the small sample size, retrospective single-center design, single time-point biomarker measurement, absence of structured symptom burden data, and lack of direct comparison with validated palliative prognostic instruments, these results should be interpreted with appropriate caution. Prospective multicenter validation studies incorporating structured symptom assessment tools, functional status measures, and established palliative prognostic instruments are necessary before AISI-based prognostication can be recommended for routine clinical implementation.

**Table 1:** Baseline Characteristics and Inflammatory Indices by Survival Status.

Characteristic	Survivors (*n* = 45)	Non-survivors (*n* = 99)
Age (years), mean ± SD	72.8 ± 11.2	76.7 ± 12.1
Male sex, n (%)	24 (53.3)	61 (61.6)
Advanced malignancy, n (%)	20 (44.4)	55 (55.6)
Charlson Index, median (IQR)	7 (5–9)	9 (7–11)
ECOG PS 3–4, n (%)	36 (80.0)	91 (91.9)
Albumin (g/dL), mean ± SD	3.1 ± 0.7	2.6 ± 0.6
**AISI**,** median (IQR)**	**1398.7 (876.4-2156.3)**	**3456.8 (2234.5-5687.2)**
SIRI, median (IQR)	4.1 (2.5–6.7)	9.4 (5.9–15.6)
SII, median (IQR)	1356.2 (845.7-2189.4)	2923.6 (1876.4-4512.8)
NLR, median (IQR)	6.4 (3.9–9.8)	12.7 (8.5–19.3)

**Table 2:** Univariable and Multivariable Cox Proportional Hazards Regression Analysis for In-Hospital Mortality.

Variable	Unadjusted (Univariable)			Adjusted (Multivariable)		
	HR	95% CI	*p*	HR	95% CI	*p*
Inflammatory Indices						
**AISI > 1850**	6.14	3.89–9.69	< 0.001	**4.38**	2.41–7.96	< 0.001
SIRI > 6.5	5.21	3.28–8.27	< 0.001	3.72	2.08–6.65	< 0.001
SII > 2000	3.94	2.53–6.14	< 0.001	2.86	1.64–4.98	< 0.001
NLR > 8.8	3.02	1.91–4.76	< 0.001	2.24	1.32–3.81	0.003
**Clinical Covariates**						
Age ≥ 75 years	1.72	1.18–2.51	0.005	1.54	1.12–2.11	0.008
Serum albumin < 3.0 g/dL	2.64	1.74–4.01	< 0.001	2.03	1.42–2.90	< 0.001
ECOG PS 3–4 vs. 0–2	2.44	1.49–3.99	< 0.001	2.12	1.34–3.35	0.001
Male sex	1.21	0.79–1.85	0.377	1.14	0.74–1.76	0.551
Charlson Comorbidity Index	1.18	1.08–1.29	< 0.001	1.09	0.99–1.20	0.082
**Model Performance (AISI model)**				**C-index = 0.81**		
				95% CI: 0.75–0.87		

Multivariable models adjusted for age, sex, Charlson Comorbidity Index, ECOG performance status (3–4 vs. 0–2), and serum albumin (< 3.0 g/dL). Each inflammatory index was modelled separately to avoid collinearity. Proportional hazards assumption verified by scaled Schoenfeld residuals (all *p* > 0.05). Concordance statistic (Harrell C-index) for the AISI-containing model: 0.81 (95% CI: 0.75–0.87). HR, hazard ratio; CI, confidence interval; AISI, Aggregate Index of Systemic Inflammation; SIRI, Systemic Inflammation Response Index; SII, Systemic Immune-Inflammation Index; NLR, Neutrophil-to-Lymphocyte Ratio; ECOG PS, Eastern Cooperative Oncology Group performance status.

**Table 3:** Univariable and Multivariable Logistic Regression Analysis for ICU Admission and Mechanical Ventilation.

Variable	ICU Admission						Mechanical Ventilation					
	Unadjusted			Adjusted			Unadjusted			Adjusted		
	OR	95% CI	*p*	OR	95% CI	*p*	OR	95% CI	*p*	OR	95% CI	*p*
**Inflammatory Indices**												
**AISI > 1850**	5.82	3.12–10.86	< 0.001	**3.96**	1.89–8.28	< 0.001	6.08	3.20–11.55	< 0.001	**4.15**	1.98–8.69	< 0.001
SIRI > 6.5	4.93	2.61–9.30	< 0.001	3.41	1.63–7.13	0.001	5.16	2.71–9.83	< 0.001	3.58	1.71–7.50	0.001
SII > 2000	3.77	2.01–7.07	< 0.001	2.74	1.32–5.68	0.007	3.94	2.08–7.45	< 0.001	2.88	1.38–6.00	0.005
NLR > 8.8	2.94	1.57–5.50	0.001	2.19	1.09–4.39	0.027	3.11	1.64–5.90	< 0.001	2.33	1.16–4.66	0.017
**Clinical Covariates**												
Age ≥ 75 years	1.58	0.89–2.80	0.118	1.42	0.79–2.56	0.240	1.64	0.91–2.96	0.100	1.47	0.81–2.67	0.206
Albumin < 3.0 g/dL	2.31	1.21–4.41	0.011	1.88	0.96–3.68	0.064	2.44	1.27–4.68	0.007	1.96	0.99–3.87	0.052
ECOG PS 3–4	2.07	0.97–4.40	0.059	1.83	0.84–3.99	0.128	2.19	1.01–4.74	0.047	1.91	0.87–4.20	0.107
Male sex	1.14	0.63–2.06	0.662	1.09	0.59–2.01	0.778	1.08	0.59–1.98	0.800	1.03	0.55–1.93	0.921
**Model Performance (AISI)**	**AUC 0.804**			95% CI: 0.729–0.879			**AUC 0.812**			95% CI: 0.738–0.886		
Hosmer–Lemeshow p	—			*p* = 0.41			—			*p* = 0.38		

Multivariable models adjusted for age, sex, Charlson Comorbidity Index, ECOG performance status (3–4 vs. 0–2), and serum albumin (< 3.0 g/dL). Hosmer–Lemeshow goodness-of-fit: *p* = 0.41 (ICU admission), *p* = 0.38 (mechanical ventilation). ROC curves for both models presented in Supplementary Figure S1. OR, odds ratio; AUC, area under the curve; CI, confidence interval; ICU, intensive care unit; AISI, Aggregate Index of Systemic Inflammation; SIRI, Systemic Inflammation Response Index; SII, Systemic Immune-Inflammation Index; NLR, Neutrophil-to-Lymphocyte Ratio; ECOG PS, Eastern Cooperative Oncology Group performance status.

## Data Availability

No datasets were generated or analysed during the current study.
